# Inter-species differences in wound-healing rate: a comparative study involving primates and rodents

**DOI:** 10.1098/rspb.2025.0233

**Published:** 2025-04-30

**Authors:** Akiko Matsumoto-Oda, Daisuke Utsumi, Kenzo Takahashi, Satoshi Hirata, Atunga Nyachieo, Daniel Chai, Ngalla Jillani, Michel Raymond

**Affiliations:** ^1^ Graduate School of Tourism Sciences, University of the Ryukyus, Okinawa, Japan; ^2^ Advanced Medical Research Center, Faculty of Medicine, University of the Ryukyus, Okinawa, Japan; ^3^ Department of Dermatology, Graduate School of Medicine, University of the Ryukyus, Okinawa, Japan; ^4^ Kumamoto Sanctuary, Wildlife Research Center, Kyoto University, Kyoto, Japan; ^5^ Kenya Institute of Primate Research, Nairobi, Kenya; ^6^ Institut des Sciences de l’Evolution, University of Montpellier, CNRS, Montpellier, France

**Keywords:** skin, wound-healing rate, humans, Cercopithecinae (cercopithecines), eccrine gland density, skin thickness

## Abstract

Injuries, which affect survival and biological functioning, are common in the animal kingdom. This study systematically investigated whether the slow wound healing observed in humans is a unique characteristic within the primate order. First, we found no significant difference in wound-healing rates between baboons under experimental conditions and those in their natural environment (0.613 mm d^−1^). Second, comparisons among four non-human primates (velvet monkeys, Sykes’ monkeys, baboons and chimpanzees) revealed no significant differences in wound-healing rates. Furthermore, these rates showed no significant differences compared to those observed in rodents, suggesting a potential commonality in wound-healing rates across diverse animal species. In contrast, human wound-healing rates were found to be markedly slower (0.25 mm d^−1^), approximately three times slower than those observed in non-human primates. This finding indicates that the slow wound healing observed in humans is not a common characteristic among primate order and highlights the possibility of evolutionary adaptations in humans. Understanding these inter-species differences in wound-healing rates may provide valuable insights into the evolutionary implications of wound healing. This study also underscores the need for further research into the biological processes underlying wound healing in various species.

## 1. Introduction

Injuries are widespread in the natural world and are often unavoidable for most animals. Generally, all animals sustain some form of injury during their lifetime [1]. These injuries cause physical damage to tissues and organs, and thus significantly impact the survival and functioning of these animals. Even though reports on injury types and their causes in wild animals are limited, it has been noted that injury is the main reason many wild mammals are brought to rescue facilities. For example, the most common reason for mammal rescue in southern Australia is collisions with vehicles (33.5%) [2]. Bats frequently experience tears in their flight membranes owing to contact with natural or artificial objects [3,4], and bite wounds are also common in terrestrial mammals, including baboons [5] and brown rats [6]. Generally, these animal injuries are often accompanied by skin wounds, hereafter referred to as ‘wounds’.

While wounds are generally not considered lethal for animals other than juveniles, secondary damage resulting from wounds, such as bacterial infections following bite wounds, can be fatal as observed in field studies on baboons [7–9]. Wounds around the mouth can decrease food intake and processing and injuries (often accompanied by wounds) that result in limping can impair climbing ability and reduce access to quality feeding areas, thereby limiting the ability of animals to access food and avoid predators [5]. Reportedly, wound healing and regeneration, which involves tissue repair and reconstruction, require energy and molecular components, such as proteins and carbohydrates. However, given the limited availability of these components, the outcome for most animals is often a trade-off between healing, growth and reproduction [10–13]. Therefore, rapid wound healing is crucial for animal survival.

Research on wound-healing rates in wild animals is very limited, and it remains unclear whether the wound-healing rates of wild animals in their natural environments are similar to those of the same species under experimental conditions. Generally, researchers often consider two methods when designing studies on wound healing [11]. The first approach involves creating wounds on animals captured from the wild or on laboratory animals and then monitoring healing in a controlled laboratory environment. This method allows for environmental control and the standardization of the wounds, facilitating comparisons between individuals. However, capturing and maintaining animals in captivity is often impractical for many species, and the stress associated with the animals being in captivity can also affect wound healing. The other approach is to observe the animals in the wild. Via this method, researchers wait for animals to sustain natural injuries and then qualitatively observe their healing.

Veterinary research has proposed that wound-healing rates vary across species, but the healing process of skin wounds begins immediately after injury and progresses through the following three overlapping stages: inflammation, proliferation and remodelling. Animals have been used as translational models to study wound healing in humans owing to their cost-effectiveness, genetic modifiability, the possibility of promoting healing and the ability to obtain tissue samples for analysis and examination. While it is generally believed that the healing process follows the same stages in all animals, the healing mechanisms differ among species [14,15]. For instance, rats, mice and ponies heal primarily via wound contraction, whereas pigs and horses, whose skins are firmly attached to the underlying biological structures, heal mainly via re-epithelialization. Furthermore, relative to ponies, horses heal at a much slower rate. Veterinarians often treat dogs and cats using the same protocols. However, research has shown that cats heal via wound contraction, showing a much slower wound-healing rate than dogs, which heal via re-epithelialization. Additionally, wound healing is faster in mice and rats than in humans, who primarily heal via re-epithelialization.

Human wound healing occurs slowly [14], and it remains unclear whether this characteristic is unique to humans or when it emerged in the course of phylogenetic evolution. To address this issue, it is necessary to first clarify whether slow wound healing is unique to humans or is a common characteristic of primates. However, studies on wound healing in non-human primates have been primarily conducted in wild baboons, and it remains unclear whether animals under experimental conditions show similar wound-healing rates as their counterparts in the wild. In this study, we aimed to address three main questions. First, we investigated whether differences in animal wound-healing rates exist between wild baboons and baboons under experimental conditions. Second, we investigated whether the wound healing rates observed in non-human primates differ from those of other mammals by comparing them to model species. Third, we compared wound healing rates among primate species to determine whether slow wound healing is a general feature of primates, including humans, or whether it can be traced back to a common ancestor shared by humans and apes.

## 2. Methods

### (a) Subject: cercopithecines

We used three non-human primates in our experiment: anubis baboons (*Papio anubis*; *n* = 6 adult males), Sykes’ monkeys (*Cercopithecus albogularis*; *n* = 5 adult males) and vervet monkeys (*Chlorocebus pygerythrus*; *n* = 6 adult males), which are all sympatric in Kenya. All the individuals were captured in the wild and maintained at the Kenya Institute of Primate Research (KIPRE). The mean body weights (mean ± SE) of the anubis baboons, Sykes’ monkeys and vervet monkeys were 23.5 ± 2.57, 4.6 ± 1.35 and 5.1 ± 1.06 kg, respectively.

The wound healing experiments were performed at the KIPRE. The animals were anaesthetized using 10% ketamine administered via intramuscular injection. First, body hair was shaved from the backs of the animals using an electric razor and a circular skin defect (diameter = 40 mm) was created. Next, the skin tissue at the shaved area was excised into the adipose tissue, creating a full-thickness wound. No silicone/plastic ring material was used around the wound. All the animals stopped bleeding immediately after the wounds were created, and each wound was promptly treated with a gentamicin-containing ointment and covered with gauze for 1 day for protection against infection. The animals were housed in separate cages, and during wound measurements, they were anaesthetized as mentioned above. Photographs of the wounds on each animal, with a scale on one side, were taken using a digital camera at intervals of 2−3 days.

Data on natural wound healing in these animals were obtained from Taniguchi & Matsumoto-Oda [16]. These data contain information on wound surface area (*S*), length (*L*) and width (*W*) from day 1 (electronic supplementary material, tables S1 and S2). Furthermore, assuming that the wounds have elliptic shapes that did not change during the healing process, wound width could be extracted from wound surface data using the relationship between *W* and *L* on day 1, i.e. a linear relationship (*W* = *aL* + *b*, with *a* = 0.121 and *b* = 3.963). Given that $S=\frac{\pi LW}{4}$, the relationship between *W* and *S* was determined as follows: $W^{2}-aW-\frac{4bS}{\pi }=0$.

Additionally, given that there are two *W* values for any given *S*, only the positive value was considered. Healing distances on day *x* were calculated from the variation of *W* between days 1 and *x*.

### (b) Subject: chimpanzees

The subjects were five adult chimpanzees (one female and four males) from the Kumamoto Sanctuary of the Kyoto University Wildlife Research Center. The animals spend their days and nights with conspecifics in outdoor enclosures and indoor rooms equipped with climbing structures, such as wooden platforms, hammocks and ropes, and other items, such as juice dispensers, small food packets and pieces of jute bags. The mean weight (mean ± SE) of the chimpanzees was 53.92 ± 11.29 kg. Photographs of naturally occurring wounds (mainly resulting from conspecific aggressive interactions) were taken at intervals of 2−7 days. The wound locations included upper limb, lower limb, back, buttocks, abdomen, face and back of the hand. The animals were awake without anaesthesia during photography, and a ruler was placed next to the wound for length measurements.

### (c) Subject: humans

A total of 24 patients comprising 14 females and 10 males were admitted to the Department of Dermatology, University of the Ryukyus Hospital, and were recruited to investigate wound-healing rate in humans. The age varied between 25 and 99 years old, with a mean and s.d. of 69.3 and 18.0 years, respectively. Following the removal of skin tumours at various anatomical locations (lower extremities, face, back and head), photographs of the skin defects were taken before secondary skin closure.

### (d) Subject: rodents

Mice (*Mus musculus*), owing to their advantages, e.g. ease to handle and maintain, rapid reproduction rate and cost-effectiveness, constitute the most widely used animal model, particularly in physiological and biochemical research. They can also be standardized for age, sex, medical history and genetic predispositions. Genetically modified mice strains have also been used to study the molecular pathways involved in wound healing and regeneration.

In this study, mouse and rat strains BALB/c (age = 30 weeks, *n* = 8; Charles River Japan Co.) and Jcl:SD (*n* = 4; CLEA Japan), respectively, were used. The animals were administered 2% isoflurane via inhalation, and thereafter, body hair was shaved from their backs, and the animals were wounded. No silicone/plastic ring was used around the wounds. After the wounding, all the animals were rapidly exsanguinated, and the wounds were monitored using a scale at 1−2 days intervals.

### (e) Healing rate

Epithelial growth is adequately described by a linear function [17,18], and the slope of this linear function represents the wound-healing rate. To avoid spatial confounding, wound healing distances were measured between opposite edges where the wound curvature was minimal. For an approximately elliptical wound, this represents the distance between the wound edges that intersect with the minor axis of the ellipse. Each measurement was independently performed two times using ImageJ software v. 1.52 a [19]. The reference wound size was measured at time *t* = 0, when the wound size was maximal. If an initial increase was observed in the wound area, as is the case at times during strong inflammatory responses [11,20], the maximum wound size was used as the reference for the wound size at other time points. The measured distance at each time point was determined as the reference wound size minus the actual wound size, and the value varied between 0 (at *t* = 0) and the reference wound size (at the time of complete healing).

### (f) Statistical analysis

Linear regression analysis was performed to assess wound-healing distances as a function of time, and changes in wound-healing distances according to species were modelled as species-specific effects on slope variation. Wound-healing distances for different specimens were measured repeatedly (i.e. each wound distance was measured twice, and each individual was considered at multiple time points). Thus, the wound healing distances were considered to be random effect variables. Generalized linear mixed models with a Gaussian error structure were used. The maximum random effects structure (intercept and slope) was employed in accordance with the recommendations of Barr [21]. Furthermore, to force the models to fit away from singularities, the Bayesian blmer function of the blme package in R software v. 4.1.2 (https://www.r-project.org/) was used, and time was scaled. The significance of the variation in deviance was determined using an *F*-test performed using the ANOVA function in the R package, car. For humans, the variation of wound-healing distance with age was modelled as the effect of the age class alone on slope variation. Three age groups were considered as follows: below or above the median and the tertile or quartile ranges. All statistical analyses were performed using R software v. 4.1.2.

## 3. Results

### (a) Comparison of healing rates between baboons in the wild and those under experimental conditions

**Figure 1.**
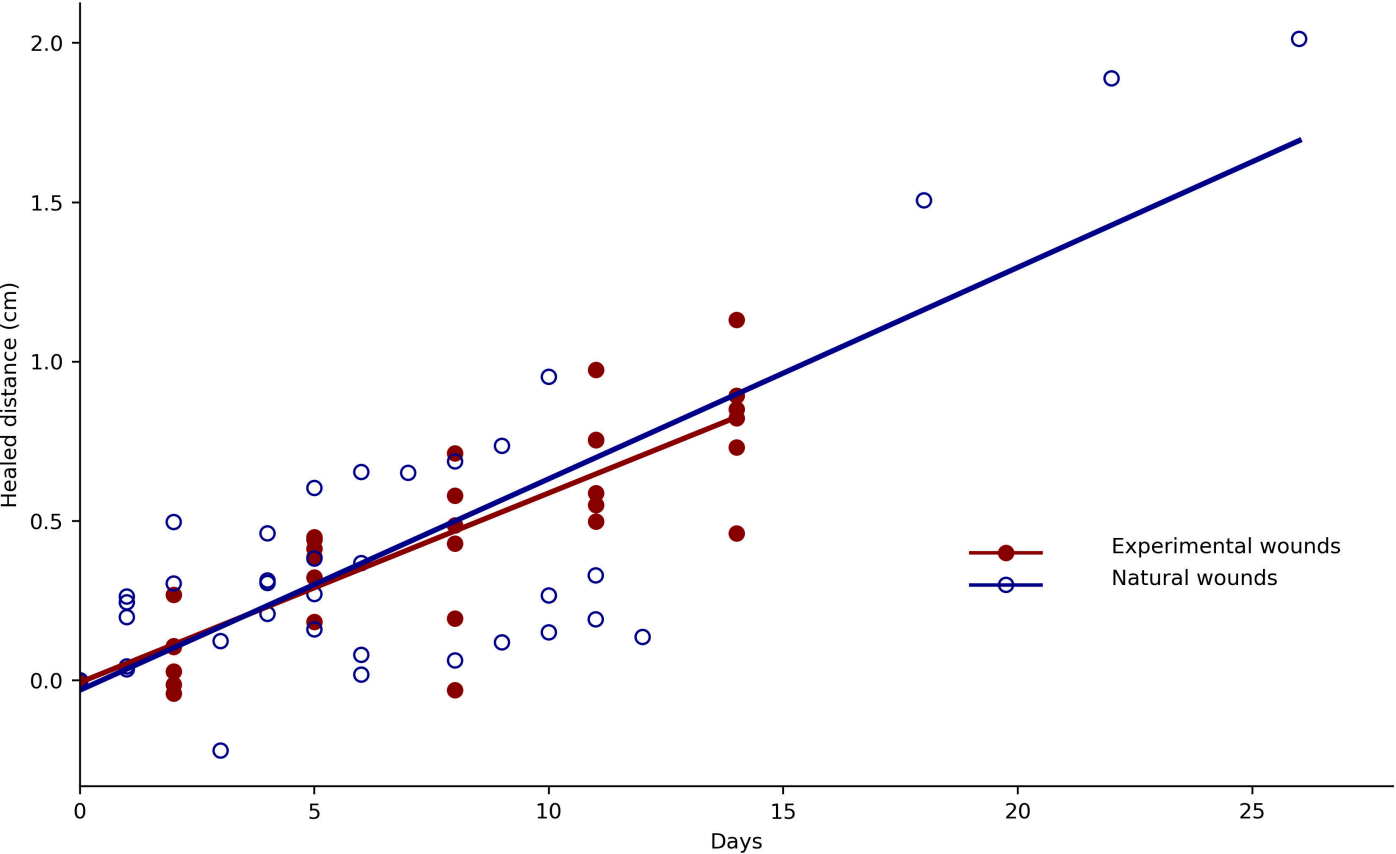
Wound-healing rate in baboons (*P. anubis*). Comparison of experimental (closed circles) and natural (open circles) wound-healing rates. For each dataset, the regression line is provided in red (experimental wounds) or blue (natural wounds). Data for natural wound healing were obtained from Taniguchi & Matsumoto-Oda [16].

A comparison of wound-healing rates between baboons in the wild and those under experimental conditions showed no significant differences between the natural wound-healing rate (0.613 mm d^−1^; SE = 0.169) and experimental wound-healing rates (slope difference: *X*² = 0.024, d.f. = 1, *p* = 0.877; figure 1; electronic supplementary material, table S1).

### (b) Comparison of healing rates between non-human primates and rodents

**Figure 2.**
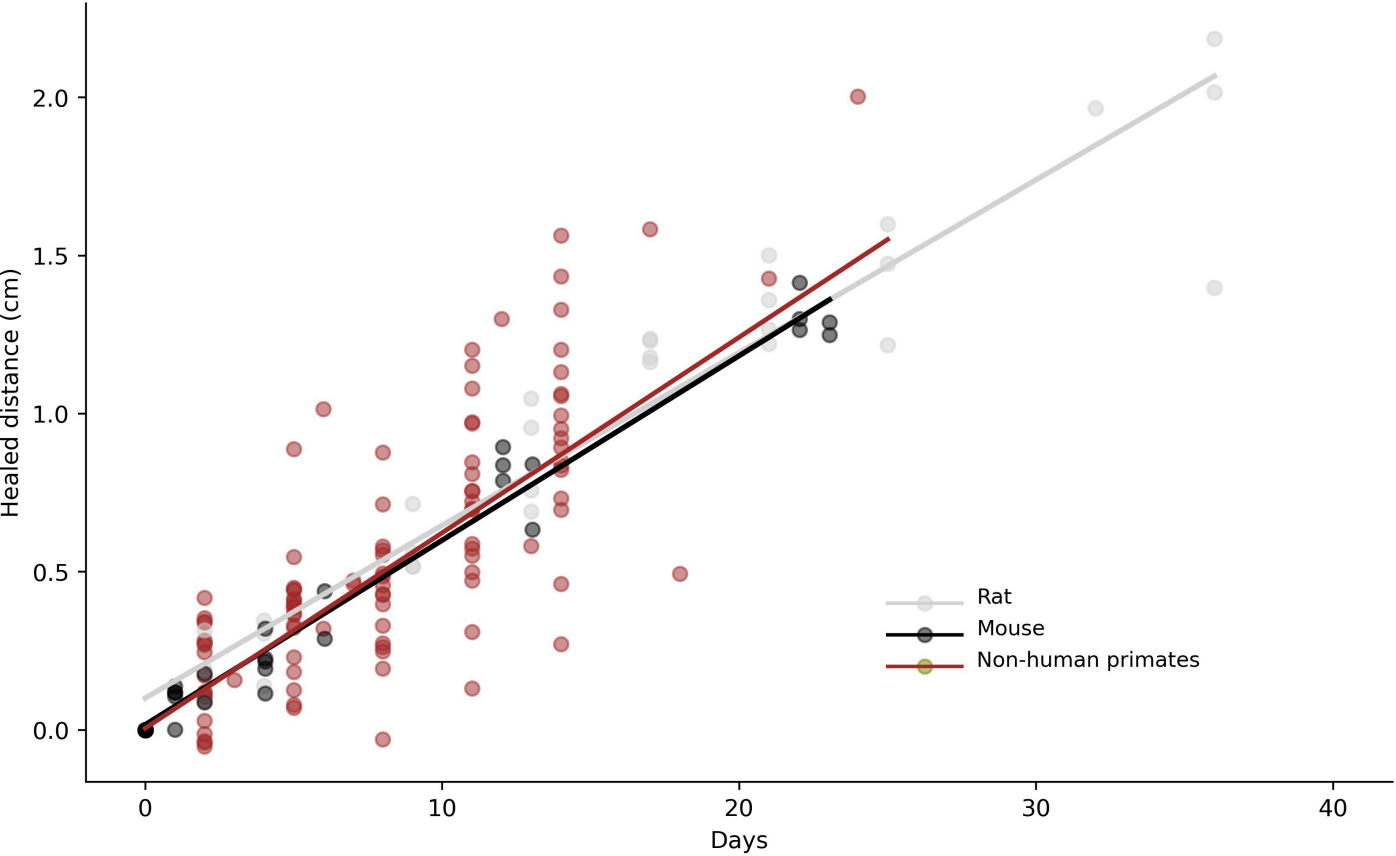
Comparison of wound-healing rate among rats, mice and non-human primates. Each dot represents the mean of two independent measurements. The regression line for each species, or group of species, is shown.

The comparison of the wound-healing rates of the four non-human primate species, velvet monkey, Sykes’ monkey, baboon and chimpanzee, with those of two hairy rodent species (rat and mouse), showed no significant differences (figure 2).

### (c) Comparison of wound-healing rates between four non-human primates and humans

**Figure 3.**
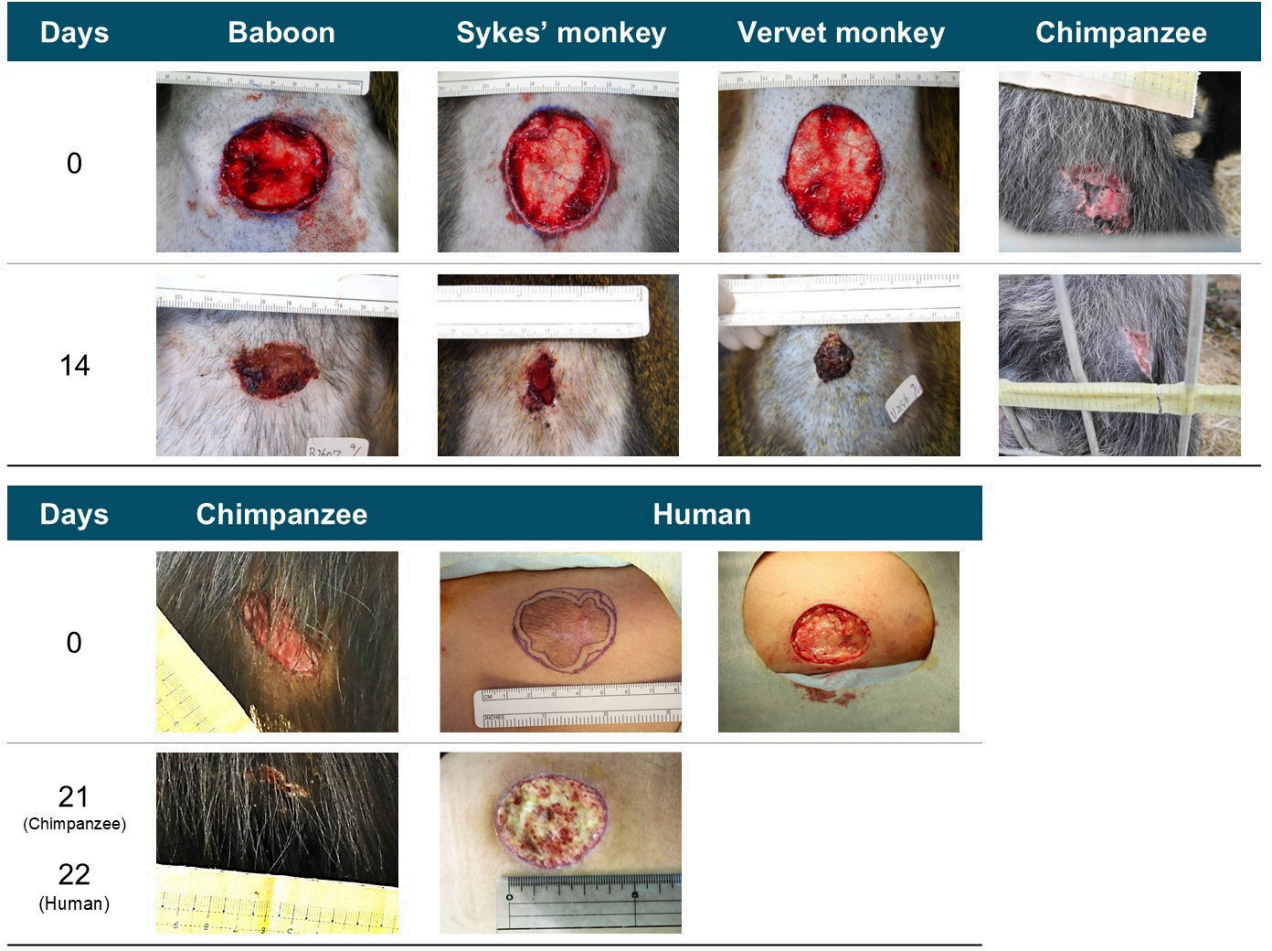
Photographs showing the progression of wound closure over a specified period. The upper row shows images from day 0 and day 14 for four non-human primate species. The lower row shows images for day 0 and day 21 (chimpanzee) and day 22 (human).

**Figure 4.**
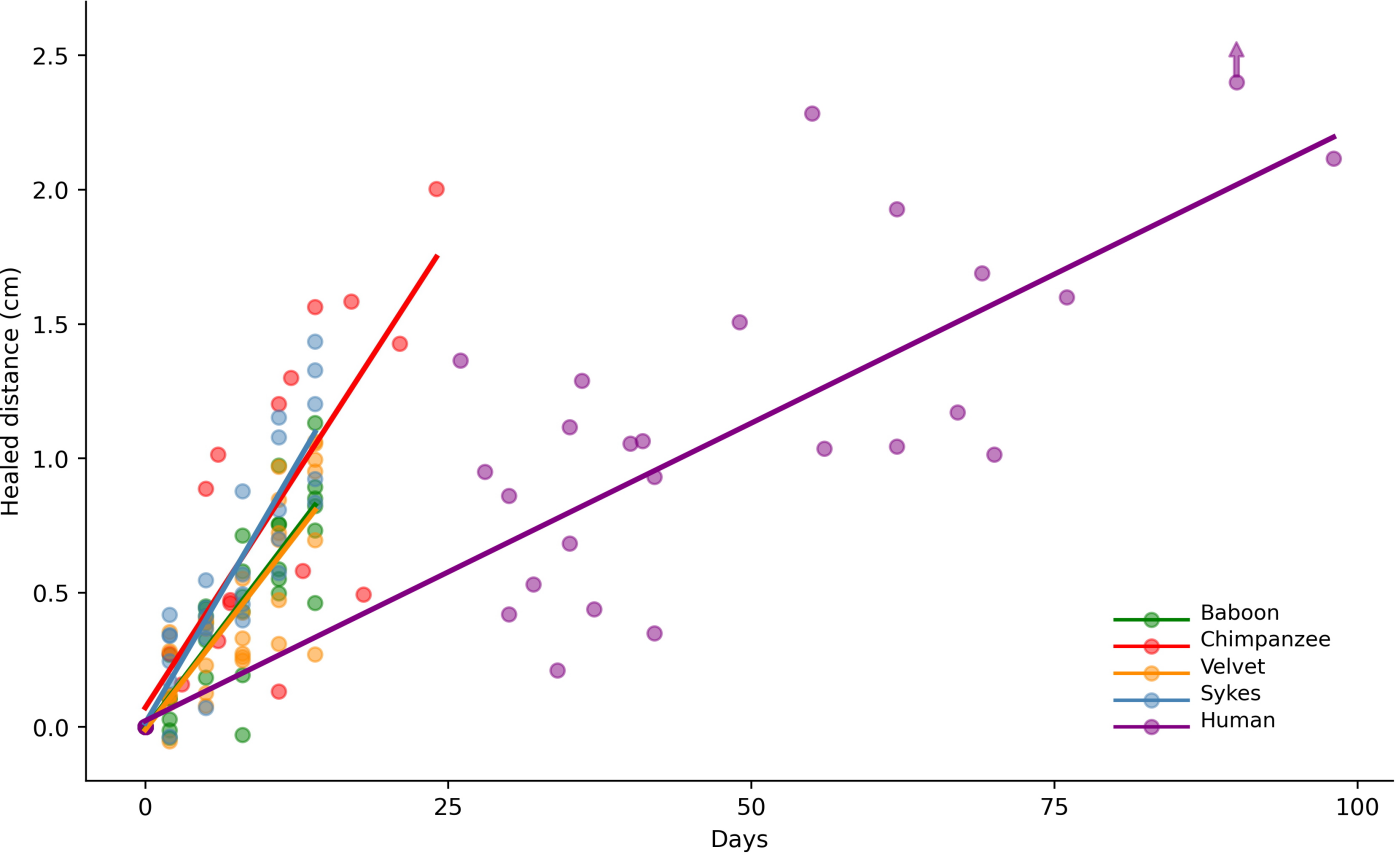
Comparison of wound-healing distances among primates. Each dot represents the mean of two independent measurements. The regression line for each species is shown. One point lying out of this graph (at *y* = 4.54 cm) is indicated using a vertical arrow.

Comparison showed no significant differences among the wound-healing rates of the four non-human primate species (velvet monkey, Sykes’ monkey, baboon and chimpanzee; *X*^2^ = 0.994, d.f. = 3, *p* = 0.803; figures 3 and 4; electronic supplementary material, table S2).

The human wound-healing rate was 0.25 mm d^−1^ (SE = 0.024), a value that was significantly lower than that obtained for the non-human primates, i.e. the wound-healing rate in humans was approximately three times lower than the mean value observed for the non-human primates (*p* < 10^–1^; electronic supplementary material, table S3). In humans, no significant wound-healing differences were observed with respect to sex (*p* = 0.79; electronic supplementary material, table S4), age (*p* > 0.73; electronic supplementary material, table S5) or wound site (*p* > 0.94; electronic supplementary material, table S6).

## 4. Discussion

This study extends previous research on wound-healing rates in mammals in three ways as elucidated below.

### (a) Comparison of wound-healing rates in baboons under captivity and in the wild

This study compared the wound-healing rates of baboons under experimental conditions with those of their counterparts in the wild, showing no significant differences in this regard. Studies on wound healing generally utilize two approaches. The first approach involves creating wounds on model animals or captured wild animals followed by the monitoring of healing under laboratory settings. The second approach involves tracking wounds sustained naturally by animals in the wild. However, concerns that healing observed under laboratory settings may not accurately reflect natural wound healing in the wild have been raised [11]. For example, in wild baboons, wounds are primarily elongated lacerations caused by canine teeth [5], while wounds created via a biopsy punch in a laboratory are circular, implying differences in wound shape, which could result in differences in wound-healing rates. Additionally, to adhere to ethical guidelines, laboratory animals may receive treatments and be subjected to cleanliness measures that are not characteristic of the wild. Regardless, this study revealed no significant differences in wound-healing rates between wild and captive settings, implying that the results obtained under experimental conditions can be applied to wild animals and vice versa.

### (b) Comparison of wound-healing rate between non-human primates and rodents

This study revealed similar wound-healing rates for four non-human primate species and rodents. Given that the skin serves as the first line of defence, protecting the body from the external environment, rapid wound healing following injury is of great biological importance. Although this study is based on a limited number of primate species, it is the first to experimentally and observationally examine wound-healing rates in multiple primate species. The results obtained indicated a common healing rate among cercopithecines, which constitute a significant portion of the primate order, and chimpanzees, which are genetically and phylogenetically the closest relatives of humans. This observation suggests that non-human primates share a common healing rate.

Present understanding of mammalian wound healing is based on the extrapolation of data from experiments involving rodents, livestock (such as pigs, dogs and horses) and humans [22]. Historically, the limited understanding gained from these studies, which focused on a few species, has been generalized to all other species without considering inter-species differences [11]. Additionally, very few wound-healing experiments have been conducted using mammals other than rodents, primarily due to factors such as the cost-effectiveness and ease of handling associated with the use of rodents. Therefore, making inter-species comparisons of wound-healing rates remains challenging in practice. The finding of this study that the healing rates of non-human primates are comparable to those of rodents may suggest the existence of an evolutionarily optimal healing rate in mammals, despite the exceptions noted in some species such as pigs [14,23].

### (c) Evolutionary delay in human wound-healing rates

This study revealed that wound healing is approximately three times slower in humans than in the four non-human primate species. The phylogenetic tree is an essential external reference for discussing morphological evolution as to when the wound-healing rate in humans became slower than that of non-human primates [24–26]. Notably, the finding that the wound-healing rate of chimpanzees—the species most genetically and phylogenetically related to humans among extant primates—is comparable to those of cercopithecines suggests that the slow wound-healing rate observed in humans had not yet been acquired at the stage of the last common ancestor of humans and chimpanzees. This observation implies that the slower wound-healing rate in humans may have evolved at a certain point within the human lineage.

The acquisition of a slower wound-healing rate can be considered an evolutionarily disadvantageous adaptation given that delayed healing may hinder access to food, reduce the ability to evade predators and cause trade-offs between energy requirements for tissue repair and other processes such as growth and reproduction [5,10–13]. Nevertheless, when and why wound healing became slower in the human lineage remains unclear and requires further investigation.

Differences in wound-healing rates are often thought to be associated with differences in the macroscopic and histological structures of the skin [23]. Factors such as the coarseness of hair follicles, the thickness of the epidermis and dermis and the wound-healing mechanism (re-epithelialization or contraction) have been highlighted as possible contributors to these differences. Even though comparative studies on the skin morphology of non-human primates are limited, examining these characteristics from an evolutionary systematics perspective may provide insights into when and why the wound-healing rate in humans became slower. Body hair serves multiple functions, including acting as a protective barrier against ultraviolet radiation, providing a physical barrier against mechanical damage and providing insulation [27–29]. While the body surfaces of many modern non-human primates are almost entirely covered by densely pigmented fur, humans are covered by fine short hair, with only a few areas, such as the eyebrows, scalp, armpits and genital region exhibiting thicker hair densities. Our wound-healing experiments in rodents revealed significantly slower healing rates for individuals without fur than for those with fur (electronic supplementary material, figure S1). If skin morphology, which is related to hair density, affects the wound-healing rate, one would expect cercopithecines to exhibit faster wound-healing rates than chimpanzees or humans, given their significantly higher hair density, approximately 2 –– 21 times greater than that in chimpanzees or humans [30,31]. However, despite significant differences in appearance, humans and chimpanzees do not show any significant differences in hair density [30–32]. The results of this study showed approximately a three times higher wound-healing rate for cercopithecines than for humans, but a similar rate for cercopithecines and chimpanzees. This observation suggested that wound healing in these species is not simply determined by hair density.

In recent years, cellular-level studies have provided detailed insights into the mechanisms of skin wound healing. A key player in this process is stem cells, which have a unique niche environment in the bulge region [33–38]. Higher stem cell counts are known to accelerate the wound-healing process. Higher hair density would be expected to increase the number of stem cells. In addition, follicles that produce thicker hairs are larger, and larger follicles are, therefore, likely to contain more stem cells [39,40]. Based on this framework, we would expect that cercopithecines with high hair density and chimpanzees with thicker hair would have more stem cells, which would explain why their healing rates are faster than those of humans. There are few tissue- and cell-level data on non-human primates, and systematic comparative studies between humans and non-human primates are still very limited. Therefore, the reason why chimpanzees do not exhibit differences in wound-healing rate compared to other non-human primates remains a matter of conjecture. A tentative hypothesis is that wound-healing rates are not directly proportional to the number of hair follicle-associated stem cells but rather depend on a threshold number of stem cells sufficient for effective healing.

The development of eccrine glands and their evolutionary adaptation, which play crucial roles in thermoregulation and brain cooling in humans, also result in trade-offs such as reduced body hair and increased epidermal thickness, which potentially contribute to the slower wound-healing rate observed in humans. From a phylogenetic perspective, eccrine sweat glands are relatively new, and in most fur-bearing mammals, they are restricted to certain sites, i.e. soles and palms. This is because the balance between bone morphogenetic proteins (BMPs) and sonic hedgehog (SHH) is fixed in each spatial region of the bodies of these mammals, resulting in either hair follicle dominance or sweat gland dominance. However, in humans, the eccrine sweat glands are well-developed over a wider area of the body surface [41–43] and have evolved to coexist with hair follicles. The distribution density of sweat glands in humans is estimated to be approximately 130−600 glands per cm². Thus, the total number of sweat glands in the human body is approximately 3–4 million, a value that is about 10 times higher than that observed in cercopithecines and chimpanzees [31,43]. This characteristic allows efficient thermoregulation via sweating, facilitates adaptations that allow prolonged activity in hot environments and enables evolutionary encephalization (‘brain cooling hypothesis’ [29,44,45]) and can be attributed to a temporal discrepancy in the BMP:SHH balance, which first promotes the production of hair follicles and then sweat glands. Recent studies have suggested that the Engrailed-1 (*En1*) gene plays a role in determining both the number of eccrine sweat glands and hair follicles [46,47]. Increased *En1* expression activates BMP signalling and suppresses SHH signalling, resulting in a mechanism that promotes sweat gland formation while suppressing hair follicle formation. Even though a higher density of eccrine glands offers evolutionarily advantages, it presents a trade-off in that humans would develop less hair, resulting in a lower hair density and an increased risk of skin exposure to environmental and physical stimuli [48]. To compensate for this vulnerability, the human epidermis may have evolved to become significantly thicker and more elastic than the interfollicular epidermis of other mammals. Notably, the human epidermis is three to four times thicker than that in non-human primates [27,49,50], implying that a thicker epidermis is associated with a slower healing re-epithelialization-based wound-healing rate [14]. Therefore, the thicker epidermis may be responsible for the slower wound-healing rate in humans than in cercopithecines or chimpanzees.

In summary, when the hair density of the common ancestor of humans and chimpanzees decreased compared to that of the common ancestor of cercopithecines, their wound-healing rates likely still remained comparable to those of cercopithecines. However, during subsequent evolutionary processes, humans, unlike the great apes, experienced an increase in eccrine gland density and a further decrease in body hair density [35], accompanied by the thickening of the subcutaneous tissue as an alternative form of internal protection. This phenomenon may have contributed to the evolution of slower wound-healing rates in the human lineage.

Additionally, even though controversial, it is possible that the development of social support for older and disabled individuals in the human lineage, as well as the use of medicinal plants, mitigated the evolutionary disadvantage of delayed wound healing. For instance, the skull of an older *Homo erectus* (D3444) at the Dmanisi site (approx. 1.8 million years ago) is almost completely toothless, suggesting that survival under such conditions probably depended on cooperative behaviours, such as food preparation and nutritional support from peers [51,52]. Similarly, the remains of a Neanderthal male, Shanidar 1, provide evidence of severe cranial trauma, the loss of the right arm (probably in childhood) and a defect in the right leg [53,54]. Therefore, his survival into middle age suggested prolonged care and assistance from group members, including prey sharing and nursing [55,56]. Traces of antibacterial plants (e.g. poplar) and salicylic acid, a compound with pain-relieving properties, have been identified in dental plaque from Neanderthals from El Sidrón. This finding indicates that Neanderthals may have possessed knowledge regarding the use of natural plants to manage pain and combat infection [57]. Such social and medical support may have helped mitigate the adaptive disadvantage of a slower wound-healing rate.

A more comprehensive understanding of the underlying causes of delayed wound healing in humans requires a comprehensive approach that integrates genetic, cellular, morphological, fossil human skeletal and extant non-human primate data. Notably, advancing research into stem cell function and the interactions between eccrine glands and hair follicles is expected to yield valuable insights into the evolution of human wound-healing processes.

## Data Availability

Electronic supplementary material is available from the Dryad repository [58].
