# Correction to ‘Inter-species differences in wound-healing rate: a comparative study involving primates and rodents’ (2025), by Matsumoto-Oda *et al*.

**DOI:** 10.1098/rspb.2025.1859

**Published:** 2025-09-17

**Authors:** Akiko Matsumoto-Oda, Daisuke Utsumi, Kenzo Takahashi, Satoshi Hirata, Atunga Nyachieo, Daniel Chai, Ngalla Jillani, Michel Raymond

**Affiliations:** ^1^ Graduate School of Tourism Sciences, University of the Ryukyus, Japan; ^2^ Advanced Medical Research Center, Faculty of Medicine, University of the Ryukyus, Japan; ^3^ Department of Dermatology, Graduate School of Medicine, University of the Ryukyus, Japan; ^4^ Kumamoto Sanctuary, Wildlife Research Center, Kyoto University, Kyoto, Japan; ^5^ Kenya Institute of Primate Research, Nairobi, Kenya; ^6^ Institut des Sciences de l’Evolution, Univ Montpellier, CNRS, Montpellier, France

*Proc. R. Soc. B*. **292**, 20250233. (Published online 30 April 2025). (https://doi.org/10.1098/rspb.2025.0233)

These differences are confirmed to be typographical errors and have no impact on the results or conclusions of the study.

Before (*incorrect part highlighted in red in the attached excerpt*):

Data on natural wound healing in these animals were obtained from Taniguchi & Matsumoto-Oda [16]. These data contain information on wound surface area (*S*), length (*L*) and width (*W*) from day 1 (electronic supplementary material, tables S1 and S2). Furthermore, assuming that the wounds have elliptic shapes that did not change during the healing process, wound width could be extracted from wound surface data using the relationship between *W* and *L* on day 1, i.e. a linear relationship (*W* = *aL + b*, with *a* = 0.121, and *b* = 3.963). Given that $S=\frac{\pi LW}{4}$, the relationship between *W* and *S* was determined as follows: $W^{2}-aW\frac{4bS}{\pi }$ = 0.

After (corrected) (*corrected part highlighted in red in the attached excerpt*):

Data on natural wound healing in these animals were obtained from Taniguchi & Matsumoto-Oda [16]. These data contain information on wound surface area (*S*), length (*L*) and width (*W*) from day 1 (electronic supplementary material, tables S1 and S2). Furthermore, assuming that the wounds have elliptic shapes that did not change during the healing process, wound width could be extracted from wound surface data using the relationship between *W* and *L* on day 1, i.e. a linear relationship (*W* = *aL + b*, with *a* = 0.121 and *b* = 3.963). Given that $S=\frac{\pi LW}{4}$, the relationship between *W* and *S* was determined as follows: $W^{2}-bW\frac{4aS}{\pi }$ = 0.

